# Society Meetings

**Published:** 1915-09

**Authors:** 


**New York and New England Association Railway Surgeons.** The twenty-fifth annual session will be held at Hotel Astor, New York City, October 21, 1915, under the presidency of Dr. W. H. Marcy, of Buffalo, N. Y. Railway surgeons, attorneys and officials, and all members of the Medical profession are cordially invited to attend. Dr. George Chaffee, Corresponding Secretary, 338 47th Street, Brooklyn, N. Y.

---

**American Proctologic Society.** Seventeenth Annual Meeting, San Francisco, June 21 and 22. The President, Louis J. Krouse, Cincinnati, in the chair. Officers elected for ensuing year: President, T. Chittenden Hill, Boston; Vice-president, Frank C. Yeomans, New York; Secretary-Treasurer, Alfred J. Zobel, San Francisco. The meeting for 1916 will be in Detroit.

President's Address, "Retrospect and Prospect." By Louis J. Krouse, Cincinnati.

Dr. Krouse stated that nearly two decades had now elapsed since the organization of the American Proctologic Society, and that he recalled distinctly the great enthusiasm manifested by the members at their first meeting.

He called attention to the fact that there is now a fair enrollment of Fellows from widely scattered parts of the United States.

He believes that the medical fraternity has still need of a society like the American Proctologic Society, whose field of activity is limited to ailments located in the anus, rectum, and colon. This is evidenced by how the general practitioner generally handles such cases, and by their later referring them to the proctologist.

Dr. Krouse deplored the fact that the medical schools are so slow in establishing a chair of proctology, such as has been done in all of the important postgraduate schools of the country, where this important branch of surgery can be taught. He thinks, and advocates, that there should be a ward set aside in all the teaching hospitals where the student will be able to acquire a better knowledge of this specialty, and will then be better prepared to treat such cases intelligently.

Dr. Krouse said that he pointed with much pride to the fact that with one or two exceptions all the best text books by American authors on the subject of Diseases of the Anus, Rectum, and Colon, have been written by Fellows of the American Proctologic Society.

A Report of Proctologic Literature, from March, 1914, to March, 1915, by Samuel T. Earle, Baltimore.

In this review of Proctologic Literature Dr. Earle quotes freely from the following authors, giving the salient points from each of their papers:

Chas. H. Mayo, M. D., Rochester, Minn., (Surgery, Gynecology, and Obstetrics, Vol. XVIII, April, 1914, No. 4, page 401), "Resection of the Rectum for Cancer with Preservation of the Sphincter."

Daniel Fiske Jones, M. D., Boston, Mass., (Boston Med. and Surg. Journal, Vol. CLXXI, July-Dec., 1914), "Cancer of the Rectum."

Joseph Weiner, M. D., New York City, N. Y., "A New Operation for Stricture of the Rectum or Sigmoid."

P. Lockhart Mummery, F. R. C. S. Eng., London, Eng., (The Lancet, Vol. 1, 1914), "Pain After Operation For Internal Piles, and Its Prevention."

P. Lockhart Mummery, F. R. C. S. Eng., and M. K. Joshi, M. R. C. S. Eng., L. R. C. P., London, (The Lancet, Feb. 13, 1915,) "Death from Strangulated Hemorrhoids."

E. Palier, M. D., New York City, N. Y., (New York Medical Journal, Jan. 23, 1915) "Hemorrhoids and Hyperchlorhydria."

Chas. Gordon Heyd, M. D., New York City, N. Y. (Surgery, Gynecology, and Obstetrics, 1914), "A Procedure for the Repair of Accidental Injuries to the Rectum."

Rectal Prolapse and Its Mechanics. By Wm. M. Beach, Pittsburgh.

The terms prolapse and procidentia are interchangeable as applied to a dislocated rectum downward on account of defective anchorage.

Dr. Beach feels assured that many of the victims of dyschezia could give a history of prolapsus in childhood.

He states that we are coming to think of prolapsus in terms of hernia, the verity of which must be determined from a consideration of the pelvic fascia and intra-abdominal pressure. He gives in detail the anatomical reasons for the causation of rectal prolapse.

Under the head of treatment he states that a number of surgical procedures have been devised and advocated for the restoration of the dislocated rectum; that they have seemed to succeed for a time, only to prove a failure later on. He mentions several of the procedures and gives his reasons for and against their employment. He gives also his operation of choice.

Cause of Dissatisfaction With Hemorrhoidal Operations. By Rollin H. Barnes, St. Louis.

The reason for dissatisfaction with the textbook methods in the operative treatment of hemorrhoids, is that they are based upon the fear of hemorrhage, and that pace has not been kept with modern surgical knowledge in regard to the control of this hemorrhage.

It is easier to take care of primary hemorrhage than of secondary bleeding such as may occur from a slough following the ligature or the clamp and cautery operation, because the surgeon is not always at hand when the latter occurs.

In the methods of Dr. J. Rawson Pennington, (A. P. A. Trans., 1914), and the author, (A. P. A. Trans., 1912), a clean excision of the hemorrhoid is done, so that it requires only controlling the primary hemorrhage, for there is no slough. The tissues are injured as little as possible so that they will retain the greatest amount of resistance against infection. There is less pain in these open methods for we do not have the "confined infection" which is especially caused by the use of sutures and by injuries to the deeper tissues.

For the control of hemorrhage the author advocates the use of pressure. Also care should be taken of the bleeding vessel itself rather than a ligature should be tied around a mass of bleeding tissues, or they should be cauterized. He also advocates that advantage be taken of that muscular contraction which can be secured to the greatest extent by minimizing trauma. The rectal plug acts against this muscular contraction.

The author opposes the customary purgation in the preparation of the patient before operation. He prefers the cold enema as a means to clean out the lower bowel. He contends that the daily enema in the after-treatment does not result in constipating the patient but rather aids in securing regularity of bowel action.

Report of Case of Carcinoma of the Sigmoid: With Stereo-Radiograms. Walter I. LeFevre, Cleveland.

Patient, male, age 55 years. Suffered with abdominal pain in the left iliac fossa for one and a half years. Complained of constipation, becoming gradually worse until a natural passage was impossible. Use of enemas resorted to but difficult to retain.

Stero-roentgenogram made by injecting Barium Sulphate emulsion (consisting of Barium Sulphate 6 oz., Pulv. Gum Tragacanth, 2 drams, Aqua, 40 oz.) This would start to be expelled when about 10 oz. was injected, but by repeated efforts 30 oz. was finally injected and retain long enough to get the pictures. Some of the emulsion passed to the upper end of the ascending colon; the transverse colon was filled; the descending partially filled; the sigmoid and rectum entirely filled. The pictures show the sigmoid loop bound down in the pelvis and almost occluded. Operation confirmed the findings. Condition hopeless. Patient died.

Emetin Hydrochloride in the Treatment of Amebic Dysentery. By Geo. B. Evans, Dayton.

Amebic dysentery is epidemic in tropical regions. It my become endemic by importation. Although various authors have contributed to a very comprehensive knowledge of the disease, there still exists considerable confusion in the interpretation of those symptoms and signs which make for accurate diagnosis and prognosis.

Dysentery may persist for months or years after the amebic ulcerations have been healed, without amebiasis being present. It may exist in a mild or severe form.

A positive diagnosis can only be made by the aid of the microscope. The smears should be taken preferably from the ulcerations on the free border of the rectal valves.

The author believes that treatment by irrigation is a thing of the past. It has been supplanted by emetine hydrochloride hypodermically.

Diet and rest are very important in treatment.

The conclusions are that what quinine is to malaria, and mercury to syphilis, emetine hydrochloride, hypodermically, is to amebiasis.

The Present Status of Local Anesthesia in the Surgery of the Lower Bowel. By Louis J. Hirschman, Detroit.

Nowhere has the real value of local anesthesia been demonstrated more conclusively than in entero-proctologic surgery.

Dr. Hirschman employs local anesthesia in the surgical treatment of the majority of his cases of anal and rectal diseases, as well as in a small proportion of cases involving surgery of the colon. The results in both classes of surgical operations have been so satisfactory to both the patient and the surgeon that the author advocates with great earnestness the further employment of local anesthesia not only in the field of intestinal surgery but also in every branch of surgical activity where absolute unconsciousness of the patient is not a strict necessity.

The technique which Dr. Hirschman uses in his ano-rectal operations and in his work on the colon is given in detail.

Which is the Best Anesthesia to be Used in Anal and Rectal Surgery. By Wm. H. Kiger, Los Angeles.

Dr. Kiger was prompted to write this paper on seeing a statement in a recently published book on "Diseases of the Rectum and Colon" which read, "Spinal anesthesia has a very limited field of usefulness. Indeed one is hardly ever justified in using it in rectal work."

After a personal experience in over five hundred rectal operations without a single unpleasant result, the writer of this paper is constrained to differ from the text-book author, and is forced to the opinion that the latter has not given spinal anesthesia a fair trial, or that he, mayhap, did not use the proper agents.

Dr. Kiger called the attention to the ease of administration of spinal anesthesia; that it may be given without assistance of an expert anesthetist; that it saves time by doing away with the delay incident on operation under a general anesthetic; that by its use the dangers of chloroform and ether are eliminated, as are also their after effects; that when it is employed there is no need to dilate the sphincters as all the operator has to do is to ask the patient to strain and the gut will easily protrude through the relaxed sphincters; and finally that it avoids shock.

He uses novocain or tropococain and gives in detail his technique for spinal anesthesia.

Further Observation on the Treatment of Pruritus Ani by Autogenous Vaccines. By Dwight H. Murray, Syracuse.

In making the fifth report of his original research work on Pruritus Ani and Pruritus Vulvae, Dr. Murray gave the results of the examinations concerning the etiology of twenty-one additional cases together with their treatment, complications, and present condition. He also reported further on the cases previously examined, treated, and reported.

Dr. Murray hoped that no reader of his papers on this subject imagined for a moment that he claimed that all of these cases made prompt and complete recovery with no relapses.

He believes that he is still justified in emphasizing the claim that most cases of Pruritus Ani and Vulvae are due to a local infection which may be benefited by treatment with autogenous vaccines.

Where he was unable to find streptococcus infection at the first bacteriologic examinations, this year when the patient had a slight relapse streptococcus fecalis was found. This gives additional evidence that infection may be present and yet the bacteriologic report not show it.

Even when we have the knowledge that it is a skin infection; that the phagocytic power of the blood is below normal for the infecting bacteria; and that the vaccine injections give the best and most lasting relief; yet we are still unable to give patient a definite statement as to the number of treatments or length of time necessary before improvement will begin. Nor are we able to assure them that no relapse will occur.

Six cases confirm the claim made in the fifth conclusion of his third report, namely: "The presence of skin infection with a local lesion begets an unfavorable prognosis for the of the pruritus ani by an operative procedure."

Three cases confirm the claim made in the sixth conclusion of his paper in 1913, namely: "The absence of a demonstrable skin infection with pruritus ani together with the presence of a local lesion will justify a favorable prognosis for the cure of the pruritus ani by an operative proceedure."

Acute cases do not seem to obtain the benefit from the vaccine treatment that chronic local infections receive.

Dr. Murray noticed that three of his very severe cases received little benefit during their course of treatment. He advised suspension of treatment and within a short time a marked improvement occurred in the severity of the pruritus and later the patients reported that the itching had practically ceased.

He states that he can account for this only upon the hypothesis that they were in a continuous negative phase while the vaccine was being administered, and that after discontinuing it they came into a positive phase. This might be taken as evidence that vaccine may be continued too long or the doses given too frequently.

The author says that in all the patients that he has examined and treated during the past year, it is remarkable that the cases of fistulae, hemorrhoids, ulcer, cancer, diseased crypts, hypertrophied papillae, constipation, and stricture gave no history whatever of having a pruritus ani. Yet authors still give these as causes. This confirms his statement in the second conclusion of the second report, namely: "Even when there is a discharge of pus or other moisture on the perianal skin it is not the actual cause of pruritus ani unless there is a streptococcic or other infection of the skin. They may exist together, but if so it is a coincidence."

This proof should satisfy the most skeptical and is an investigation that all can make without trouble.

The relapsed cases that returned for treatment have responded more readily to the vaccine treatment than when they first came, and some who have not returned report that the itching is easily controlled.

Results of treatment by autogenous vaccine still continue to be the most satisfactory of any Dr. Murray has yet used. Patience and perseverance are necessary on the part of both the patient and the physician.

Peritoneal Adhesions and Intestinal Stasis. By Jas. A. MacMillan, Detroit.

The author of this paper states that the interest of the medical profession in this subject was awakened by the work of Mr. Arbuthnot Lane, of London, England; that there is a demand for operative interference in many cases of intestinal stasis, but for an operation less radical than that of extirpation of the colon; that although in the majority of instances they are not causative factors, peritoneal adhesions in some instances produce intestinal stasis.

He further states that there are two points in diagnosis which the paper is intended to emphasize: (1) The importance of pain and tenderness. (2) That the offending adhesion will be found to belong to a few definite types.

Constipation With Special Reference to Its Treatment. By Lewis H. Adler, Jr., Philadelphia.

Dr. Adler called attention to the fact that the intestinal track is the chief sewer way of the body, and as such required as much attention as the plumbing in one's dwelling; that the term "Constipation" is a relative one and the line of demarcation between what is physiologic and that which is pathologic, in a given case, can only be drawn by a thorough study and knowledge of the individual; that the standard of health in one person, whose bowels move only on alternate days may be as perfect as in the individual who has two normal bowel actions per diem; that one of the chief etiologic factors in producing, or inducing, this malady is the neglect, frequently repeated, to respond promptly to the calls of nature; and to the pernicious practice which Americans, at least, have fallen into, of resorting to the taking of purgative medication.

Attention was called to the contra-distinction between obstipation and constipation. In constipation we have to deal with functional diseases of some portion of the intestinal track; while in obstipation there is normal functional activity, but there is some deformity, growth, constriction, flexion, or foreign body in the intestinal canal which offers a mechanical obstruction to the passage of the fecal current. He stated that these distinctions must be borne in mind, for while they may present similar symptoms, the treatment is entirely different.

The chief object of the paper was to lay stress upon the treatment of constipation by other than medicinal means.

The author advises that all conditions, general or local, which interfere with the health of the individual, should be removed; and that diet and hygiene should be given careful consideration. He also advises that where massage is given it should be carried out by the physician himself and not by a masseur.

The Ultimate Nervous Results of Acute Angulation of the Sigmoid, and the Consequent Fecal Stasis. Wm. H. Axtell, Bellingham, Wash.

Dr. Axtell divides the nervous end results into three general types: (a) Severe type: Including acute mania. (b) Moderately severe type: Including melancholia, chronic sciatica, chronic lumbago, tropic corneal ulcers. (c) Mild type: Including eczema, the apathetic, the neurasthenic.

He was not prepared to say whether or not the angulations found were the cause of the fecal stasis, or the stasis the cause of the acute angulation. These conditions, however, were found in all of those cases, and the nervous conditions which were produced disappeared upon correction of the angulation and stasis. Dr. Axtell's conclusions are:

(1) Many cases treated as typhoid fever are simply cases of constitutional and systemic infection from putrefactive toxins of the alimentary canal.

(2) If the true condition were recognized at the outset, and if the colon were thoroughly cleansed of the soil for the growth of typhoid bacteria, there wiuld be fewer cases of typhoid fever.

(3) Physicians do not as a whole examine the rectum and colon with the same degree of precision that they do other parts; they do not have a true appreciation of its importance; nor do they comprehend what persistence is required to empty the colon.

(4) We are all too much inclined to cling to precedent, rather than to act according to the conditions found.

Notes on Rectal Fistula. By J. Rawson Pennington, Chicago.

The etiology, conformation and classification of fistula as presented in our textbooks is not satisfactory.

The rectum extends from the termination of the sigmoid, at a point opposite the middle of the third sacral vertebra, to the pectinate line. The anal canal extends from this line to the anus. A fistula, then, with the external opening in the anal canal should be classified as an anal-fistula, or fistula-in-ano. A fistula with the internal opening in the pectinate line, or junction between the rectum and the anal canal, partakes of both of these structures, the rectum and anal canal, and should be known as an ano-rectal fistula. While those cases with the internal opening in the rectum should be known as rectal fistula. Complex, compound, horse-shoe, and other so-called varieties of fistula are simply expressions of complexity, position, or shape, of one or the other of the foregoing divisions, or a combination of them.

Methods of Treatment. Many methods have been proposed for the treatment of fistula. The author desires to submit herewith another which he believes to be far more important than any as yet presented. The Preventive Treatment. All methods may be classified under three general heads, viz.: the preventive, palliative, and curative. Under the former may be considered the prophylactic and the abortive treatment. Under the latter the injection and the operative treatment.

(a) Prophylactic Treatment.—It is said that an ounce of prevention is worth a pound of cure. This injunction is as apropos in the treatment of fistula as in the treatment of any other malady. A complete history and careful examination usually elicits the fact that practically every individual who has fistula has or has had hemorrhoids, cryptitis, fissure, pruritus ani, proctitis, or some other form of curable rectal disease. These conditions favor the invasion of the peri-rectal tissues with pyogenic organisms, which is usually followed by an abscess and fistula. Hence, if peopl [illegible] were educated to keep their rectums in a healthy state, and [illegible]lid so, fis[illegible]la would become less frequent. Since the number of cases may be reduced by education, it becomes our duty as proctologists to launch a campaign for the prevention of this loathsome affliction—Fistula.

(b) Abortive Treatment.—The time to abort fistula is during the infection or abscess stage. If the abscess is opened early and the pus allowed to escape, and the abscess wall is not interfered with, in any way with instruments or drugs, but the cavity drained freely, and gently filled with subnitrate of bismuth ointment, and this treatment repeated every two, three or four days according to the indications fistula will, as a rule, be aborted.

Fecal Abscess in Pouch of Douglas, Following Typhoid: Report of Case. By Alfred J. Zobel, San Francisco.

The author of this paper stated that for the past thirty years very few cases of fecal abscess have been reported in the literature. Only one of the more recently published text books of surgery gives even brief mention of the subject.

A fecal abscess is distinctly different from an abscess in which the pus has been so tainted by a growth of colon bacillus that from the odor it may be mistaken for fecal matter.

It may occur in connection with any portion of the intestine, and originate either externally or from within. When it originates without it may subsequently burst into the gut, empty its purulent content, and have it replaced wholly or in part with fecal matter.

A fecal abscess which originates from within the gut usually results from a slow, progressive ulceration of the mucosa, due either to general conditions, such as typhoid fever, dysentery, tuberculosis, or cancer, or to local causes, such as chronic intestinal catarrh, stricture, a hard fecal accumulation, or a foreign body.

The writer of this paper reports a case of fecal abscess which not only filled the cul-de-sac of Douglas, but also had invaded the tissues between the rectum and the vagina. The patient, a woman of forty-two years had had a miscarriage eight years previously, and was told at that time that some kind of a swelling could be felt in her rectum. However this gave her no trouble then, nor subsequently, and it had been entirely forgotten. When her present trouble began, two and a half months after an attack of typhoid fever, the history of this former condition complicated the diagnosis. On digital examination a large, smooth, immoveable, brawny mass, beginning about 2½ c. m. above the internal sphincter, and extending beyond reach of the finger, was felt bulging from the right-lateral and anterior sides of the rectum. The mucosa was freely moveable over it. No sign of fluctuation could be elicited. No particular pain was caused by deep pressure. The temperature and pulse rate were normal. It had been aspirated through the rectum by her physician, and a slightly turbid fluid had been withdrawn. The mass began to swell into the vagina, and in two days so occluded the passage that it almost prevented the entrance of the examining finger beyond the portal. Slight fluctuation was then felt. There was severe rectal pain. The temperature was still normal; the pulse 90. An exact diagnosis was not made before the operation. An incision was made through the postero-lateral vaginal wall. Upon blunt dissection a tense sac presented. When this was punctured the contents gushed out in a thick sluggish stream which kept flowing for some little time. From its strong fecal odor and brownish-yellow, lumpy appearance it apparently consisted wholly of semi-liquid, mushy feces, similar to what is found in the lower end of the ileum and cecum. Nearly two pints of this foul material was evacuated. Fecal drainage ceased entirely eight hours after the abscess was opened. The turbid discharge which remained rapidly decreased in quantity and in less than four weeks after the fecal abscess was evacuated, the wound was completely healed.

Dr. Zobel said that although a fecal abscess is met with so rarely the possibility of it being present should be taken into consideration in the differential diagnosis of obscure intra-abdominal tumors. He concluded by quoting from Fenwick: "Where there is a localized abdominal swelling, immoveable by the respiration or by a moderate amount of pressure of the fingers; whose size and shape alters when diarrhoea occurs; in which light percussion gives a tympanitic, and a more forcible stroke a dull sound; or in which an emphysematous sensation is communicated to the fingers, or a gurgling sound produced by percussion; it will be probably of fecal origin; and this more probably when there is a history of anything apt to produce ulceration.

Ischiorectal Abscess in Nine Day Old Infant: Report of Case. By Alfred J. Zobel, San Francisco.

The abscess was first noticed on the ninth day after the birth of the child. No cause could be discovered for its formation. It was incised on the twelfth day without any anesthesia. It was curious how the abscess cavity, which was quite large, filled up so rapidly, complete healing taking place at the end of a week.

---

### Report of the 40th Annual Meeting of the Alumni Association Medical Department, University of Buffalo.

The 40th annual meeting of this association, which was duly incorporated in 1875, took place Tuesday to Friday, June 1 to 4, 1915. All sessions with the exception of the annual alumni dinner, the Class and Frat reunions, were held in the college building. In point of attendance, importance and timeliness of subjects discussed at the scientific sessions, in interest and enthusiasm displayed by the alumni, the entire meeting was very successful.

SCIENTIFIC PROGRAM

**Wednesday Morning, June 2nd.**

Doctor John L. Butsch, Assistant Professor of Pharmacology, gave an interesting paper on **The Process of Testing Kidney Function by the Retention Method.** He outlined this method showing its advantages in the clinical investigation of diseases of the kidney and demonstrated the procedures used.

Dr. F. H. Pratt, Prof. of Physiology, who has in his three years' connection with our school proved his ability as a teacher and investigator, and who has also studied very extensively the problems of medical pedagogy, discussed **Modern Principles in Medical Teaching.** He stated that the main purpose of a medical school must center in the student, and the student should be judged first by the criterion of industry. The great advance made generally in this country through higher entrance requirements promises to have one regretable feature, in that the more mature student, whose attitude toward his work is so often a stimulus and fine influence, is liable now and in the future to be ruled out, owing to the obstacle of the year or two of preparation. Industry and high purpose must be fostered in the younger class of entering students by early insistence on standards.

Method of work and thought should be taught, as well as subject matter. To get the student to grasp principles and to solve problems should be the end of our teaching—a requirement as a rule very different from the sort of studying he has been accustomed to. The teacher should endeavor constantly to lead the student to think hard, and needs to verify constantly the results of his teaching. Inculcation is not enough. Verification is equally important.

Unless the teacher meet the student in the presence of nature—the actual phenomena of health and disease—the teaching is liable to lack an indispensable element. To make this possible, a large staff is needed, small sections, and pedagogical training.

Medical text-books are, as a rule, unfitted by their bulk and arrangement for class-room work. A great need in medical education is concise texts suited to the needs of systematic assignment of lessons and of recitation. Such generally do not exist.

We do not make the use of books a sufficient end in the training of students. Only the graduate who can use a library, and can refer to the literature of a subject, can be expected to progress in medicine. Such ability should be made a prerequisite to graduation.

Endowment of all higher education is becoming increasingly necessary with the rising standards of equipment and teaching. This is especially true of medical schools. Effective and smooth-running organization, as simple as circumstances permit, is equally necessary. The student must be judged in the light of elaborate and carefully preserved records, and likewise must be known as a man to many of his teachers. As the institution is judged by the student product, so must the fruits of the student's industry and character be weighed by every means in our power before he becomes a product.

Our fellow alumnus, Dr. Raymond F. Metcalfe, 1900, Major Medical Corps, U. S. A., who has been in the service for the past decade, gave a very interesting discussion of the **Organization of the United States Army with Special Reference to the Medical Corps.** He described the organization of the army in time of peace and war, outlining fully the auxiliary forces and the various units of which the army is made up. He detailed the equipment of a field hospital and the question of military sanitation and military surgery, the Red Cross service, and the different duties of the medical officers in relation to sanitation, care of the sick and wounded, the drill of hospital corps, and the treatment of epidemic in towns situated in the war zone. He mentioned that there were a few vacancies in the medical corps and hoped that some of our fellow alumni might fill same as there are now about a dozen U. of B. graduates in the service.

Dr. W. L. Moss, Internist to the State Institute for Malignant Diseases reported a **Study of over 200 Diphtheria Bacillus carriers and their relations to the community.** This was based upon investigations carried on in the city of Baltimore during a diphtheria epidemic and he showed that the presence of diphtheria bacillus (the non-virulent type) does not render the carrier a menace to the community. He cited statistics to prove his contention. Dr. William G. Bissell, '92, in discussing the paper, took exception to the guinea pig test for virulency of the Diphtheria organism.

In demonstrating **Radium and Radioactive Substances,** Dr. Otto Buseck, of the Radium Limited, New York City, showed specimens of pitch-blend, Carnotite and Fergusonite, the principal ores from which radium is extracted. He described the method of extraction and the process resulting in the formation of radium chloride, which is converted into the bromide and sulphate,—the by-products being the radioactive substances. He showed radium emanations, an activator which can be usd to charge water, radium compresses, radium gloves and radium linen, describing their therapeutic uses in every instance. In a small lead tube he had a glass vial containing 15 mg. of radium sulphate valued at about $20,000.

**Wednesday Afternoon, June 2nd.**

The **Annual Alumni Oration** was given by Dr. William House, '95, who came from Portland, Oregon, to attend our meeting. Dr. House is Assistant Professor of Neurology and Psychiatry in the University of Oregon and is a force and factor in medicine on the Pacific Coast. He is also one of our most enthusiastic and loyal alumni. His topic was **Cerebral Localization** which he treated very practically and clearly always having in mind its value to the general practitioner. He described the blood supply to the brain, and showed how the different lobes are supplied with the significance of the changes resulting from the circulatory disturbances in the various parts. He cited a novel method of remembering the position and relation of the cranial nerves,—this method being originated by the doctor himself. Such topics as Cerebral Thrombosis, Apoplexy, Brain Tumors, were fully and clearly dealt with, both as to formation and localization. Dr. James W. Putnam, '82, discussed the paper.

**Thursday Morning, June 3rd.**

Prof. House gave an unusually interesting **Neurological Clinic** in which he demonstrated, side by side, a case of Tabes and Spastic Paraplegia. He discussed the luetic origin, the pathogenesis in the respective portions of the cord, the symptomatology resulting therefrom and laid special stress on the diagnostic features which differentiated the respective diseases. A case of motor aphasia was presented and discussed with special emphasis on alcohol as a factor in arterio-sclerosis of the cerebral vessels. The various possibilities in the lesions producing the aphasias were fully stated before coming to the definite condition in this case. A case of haematomyelia was then presented, the history read, the lesion discussed with its symptoms, a differential diagnosis made between this condition, spinal hemorrhage, fracture, dislocation and tumor. Dr. George A. Sloan, '98, provided the cases from his service at the Erie County Hospital.

Dr. George W. Crile, the well-known surgeon and investigator, and Professor of Surgery in the Western Reserve University, whose researches in surgical shock, internal secretions and allied problems have been of the greatest service to modern medicine, gave an intensely interesting and illuminating surgical diagnostic clinic in which he demonstrated cases of goitre of different kinds and severity. He discussed the etiology, the symptomatology, the various diagnostic features of each case, commenting on the prognosis, and showed the relation between fever and its effects and hyperthyroidism and its results. The material for clinic was arranged by Dr. Fred Parmenter, '03.

**Thursday Afternoon, June 3rd.**

Prof. Samuel J. Kopetzky, of New York City, whose investigations in the problem of Meningitis and its otological relations have placed him among the leaders in his specialty, opened the afternoon session with a paper on **The Inter-relationships of General Medicine and Surgery to Otology, with special reference to Intra-Cranial Diseases.** He showed the importance of the study of the cerebro-spinal fluid, the relation of leucocytosis to both ascending and descending polynuclear leucocytes, and the value of careful study of the symptoms alongside of laboratory findings. The paper was profusely illustrated with lantern slides, clinical charts and tables formulated from his extensive study and experience in Otology.

Dr. Joseph C. Beck, Prof. of Oto-laryngology, University of Illinois, Chicago, an authority in his special field of activity, gave a **Borderline Clinic** in which he presented a case of goitre removal in which patient was aphasic on turning head to left, but no speech defect with rotation to right. The symptom was due to pressure of the enlarged thyroid on the left recurrent laryngeal nerve, the operation resulting in complete cessation of symptom; a case of facial palsy (traumatic in origin) in which anastamosis of the seventh nerve to the hpyoglossal was successfully performed; a post-radical facial palsy not operated; a case of cancer of the tongue involving the right half, operated on seven years ago with no recurrence and with practically no defect in speech discernible; a case of post-nasal fibro-sarcoma of seven years' duration extending from the left side of the naso-pharynx to the right in which patient refused operation; a case of tubercular cervical adenitis extending from the mastoid down to the opening of the thorax, successfully operated upon; a case of calcareous brain tumor in the left temporal region with little localizing symptoms, diagnosed by a radiograph plate which was shown and a case of chronic non-suppurative labarynthitis, after which Prof. Beck showed a series of carefully prepared lantern slides illustrative of the various borderline operations and the steps and surgical procedure of same. The clinic was arranged by Dr. Chester Cott, '08.

Dr. William House discussed papers of Professors Kopetzky and Beck, after which a special vote of thanks was given the speakers by our association.

Dean Herbert U. Williams, '89, Professor of Pathology and Bacteriology gave a most interesting demonstration of **Serum Reactions used in Modern Diagnosis.** The principal types of reactions were demonstrated in large cylinders so as to be plainly visible; precipitins; hemolysins, agglutinins all prepared by Miss Jacobs, also the phenomenon of anaphylaxis on guinea pigs, and Malone's recently published modification of the Abderhalden principle, the latter by Dr. Nadina R. Kavinoky, '10.

**Demonstrations in Physiological Laboratory.**

In his laboratory Prof. F. H. Pratt gave an interesting **Physiological Demonstration** designed to show, first, a very simple method of demonstrating to a class the auricular and ventricular beat of a small, excised mammalian heart; secondly, to illustrate a possible method of cardiac nutrition from the interior of the heart cavities.

Defibrinated blood is introduced into the right ventricle through a cannula tied into the pulmonary artery, the venae cavae having been ligatured. If the open cannula is now supported in an upright position, and the heart kept warm by dipping occasionally into warm normal salt solution, beating of right auricle and ventricle may be maintained for several hours. The preparation may be readily handed about among a class of students. Both block and fibrillation may often be observed under this method.

It is presumed that the heart walls, especially when thin, as in a small heart, may, under conditions of greatly decreased peripheral resistance, receive effective nutrition, by this means, through the endocardium; partly by direct entrance of blood through the Thebesian foramina, partly by diffusion of the soluble non-protein constituents through the endocardial lining.

ANNUAL BUSINESS MEETING.

The annual business meeting was called to order on Wednesday afternoon by President, Dr. George F. Cott, '84. In submitting the annual report of the Executive Committee, Dr. L. Kauffman, Chairman, stated the various activities undertaken during the past year in developing the spirit of loyalty and enthusiasm among the alumni of the Medical and other departments of the University. There were organized two new District Branch Alumni Associations, viz., the Chautauqua District Alumni Association at Jamestown on November 18, 1914, comprising the alumni of all departments resident in the counties of Chautauqua, Allegany and Cattaraugus of New York State, and the adjoining counties in northwestern Pennsylvania, and the Central and Northern New York Alumni Association at Syracuse, N. Y. on February 10th, 1915. In addition both the Interstate Alumni Association and the Rochester

District Alumni Association held their second reunions respectively at Elmira and Rochester.

For the first time in the history of our University the Alumni of all departments gathered together on last University Night (February 22d, 1915) and organized THE ASSOCIATED ALUMNI OF THE UNIVERSITY OF BUFFALO at a dinner held at the Hotel Statler. It was unanimously desided to form a permanent organization of all the alumni of the U. of B. and make **University Day** a practical demonstration to our alumni and the citizens of Buffalo and vicinity of what a real force and power our University may be.

With reference to the Alumni catalogue the committee reported that the catalogue was compiled by Miss Emma L. Chappell, well known to our members for her twenty years' activity as college secretary, and is now in the printer's hands. The publication, however, has been deferred as it was deemed advisable to incorporate therein the Constitution and By-Laws of our Association to be adopted at this meeting. In addition the committee has compiled a new card index of the entire membership of this association. The expense of the catalogue and card index is being defrayed from a fund raised by an equal voluntary contribution from a number of our alumni, a list of whose names will be sent with the catalogue which will surely be delivered in October.

For the furtherance of the alumni activities the following resolutions were recommended by the Executive Committee and unanimously adopted:

1. That the Alumni Association of the Medical Department respectfully suggest to the Associated Alumni, the Faculties, and Council of the U. of B. to make our "University Day" an event in our University's life each year. That the public address in the morning be continued, that the students of all the departments be jointly entertained at a luncheon in the afternoon to be given by the combined Faculties, thereby rendering possible the development of alumni spirit during the student days, and that the annual dinner under the auspices of the Associated Alumni round out the day's festivities.

2. That your executive committee be empowered to organize in conjunction with the other departments and under the auspices of the Associated Alumni a METROPOLITAN ALUMNI ASSOCIATION, comprising the alumni of all departments residing in New York city and vicinity.

3. That the Executive Committee be empowered to arrange for the settlement of all outstanding dues of our members, if such be deemed feasible the coming year.

4. That steps be taken to establish the ALUMNI FUND as provided in the newly adopted constitution.

### CONSTITUTION AND BY-LAWS

The amended constitution and by-laws were adopted and contain such provisions as will permit our Association to assume larger and more important activities and afford each and every alumnus an opportunity to become actively interested in his Alma Mater. The management of the association is delegated to a Board of Directors consisting of the president, the five vice-presidents, secretary, treasurer, the Executive Committee and Board of Trustees. As at present, the executive committee has direct charge of the general welfare of the association and consists of a chairman and of two members elected annually by the association, the president, secretary and treasurer ex-officio and one member elected from the faculty. Provision is also made for the establishment of an **Alumni Fund** which is to be raised and managed by the Board of Trustees. The control of this fund is at all times vested in the association and the expenditure of same is to be determined by the association at an annual meeting,—the interest only to be used for such purpose or purposes as will be decided upon by the association for the benefit of our Alma Mater.

The officers for the ensuing year were elected as follows:

President, Lesser Kauffman, '04, Buffalo, N. Y.

First Vice-president, Charles D. Aaron, '91, Detroit, Mich.

Second Vice-president, Archer D. Babcock, '93, Syracuse, N. Y.

Third Vice-president, James E. King, '96, Buffalo, N. Y.

Fourth Vice-president, Jane W. Carroll, '91, Paterson, N. J.

Fifth Vice-president, Lee A. Whitney, '01, Rochester, N. Y.

Secretary, Julius Richter, '04, Buffalo, N. Y.

Treasurer, Frank E. Brundage, '09, Buffalo, N. Y.

Trustee for Five Years, George F. Cott, '84, Buffalo, N. Y.

Executive Committee—Wm. F. Jacobs, '08, Chairman; Edith R. Hatch, '06; Wm. G. Bissell, '92, all of Buffalo.

Next year our officers will be elected by ballot with two tickets in the field. The opportunity will thus be afforded to every Alumnus in good standing, i.e. who has paid his dues to date, to vote by mail if unable to attend the annual business meeting in person.

During the past year we had thirty-five deaths in our ranks as per **Necrology List** compiled by Secretary Richter.

Class of

1851 JOSEPH O. ALLEN, died in Fayette, Ohio, May 20th, 1915. Age 85.

JUSTIN E. ELLIOTT, died in Ardmore, Pa., April 8, 1915. Age 87.

1860 JOHN E. BRADY, died at Grand Rapids, May 18, 1914. Age 72.

O. B. WEBSTER, died in Vassar, Mich., April 30, 1914. Age 87.

1864 WILLIAM H. GAIL, died in Buffalo, February 6, 1915. Age 74.

JOHN MASON BROWN, died in Westfield, N. Y., July 12, 1914. Age 72.

1866 HENRY W. CALDWELL, died in Pulaski, N. Y., Feb. 24, 1915. Age 73.

ROBERT T. MENZIE, died in Caledonia, N. Y., Apr. 20, 1915. Age 81.

1869 JOHN H. TAYLOR, died in Holley, N. Y., Sep. 23, 1914. Age 70.

1870 STUART H. BENTON, died in Brooklyn, N. Y., Mar. 2, 1915. Age 69.

1871 JOHN E. McTAGGART, died in Detroit, Mich., May 12, 1915. Age 76.

OSCAR S. PRATT, died in Buffalo, N. Y., July 25, 1914.

1873 ANDREW J. JACKSON, died in Mattawan, N. J., Nov. 17, 1914. Age 72.

1875 WILLIAM L. ALLEN, died in North Tonawanda, N. Y., Feb. 17, 1915. Age 67.

FREDERICK C. BEALS, died in Salamanca, N. Y., July 25, 1914. Age 62.

1876 EDWIN T. M. HURLBUT, died in Yountville, Cal., Apr. 14, 1915. Age 86.

1877 EDWARD ECKERSON, died in Denver, Col., Dec. 14, 1914. Age 61.

CHARLES SHUPE, Died in Attercliffe Station, Ont., May 6, 1915. Age 68.

1878 DANIEL E. FULLER, died in Hastings, Mich., May 12, 1915. Age 58.

1879 FLOYD B. PARKE, died in Elmira, N. Y., Oct. 9, 1914. Age 58.

1881 CHARLES B. SCOTT, died in Knoxville, Tenn., Jan. 27, 1915. Age 57.

1881 ALBERT M. COOK, died in Pasadena, Cal., Aug. 16, 1914. Age 59.

1884 WILLIAM A. OLIVER, died in Canandaigua, N. Y., Apr. 6, 1915. Age 67.

RANSOM F. ROWLEY, died in Portville, N. Y., Aug. 6, 1914. Age 57.

1887 EUGENE A. SMITH, adjunct professor of clinical surgery in his Alma Mater; attending surgeon to the Buffalo Hospital Sisters of Charity; consulting surgeon German Deaconess Hospital, Riverside Hospital, Woman's Hospital; died at his home in Saranac Lake, N. Y., Aug. 9, 1914, from pulmonary tuberculosis. Age 49.

1888 CLAYTON A. BUTTON, died in Holland, N. Y., Feb. 24, 1915. Age 61.

1890 FREDERICK E. McCLELLAN, died in Canandaigua, N. Y., Mar. 27, 1915. Age 46.

WILLIAM L. HUNTER, died in Brooklyn, N. Y., Apr. 22, 1915. Age 65.

MATTHEW J. O'CONNELL, Niagara University, died in Buffalo, N. Y., Aug. 4, 1914. Age 52.

1893 WILLIAM H. ANNOWSKI, died on his yacht near Crystal Beach, Ont., Aug. 1, 1914. Age 61.

1894 EDWIN E. KOEHLER, Niagara University, died in Buffalo, N. Y., Jan. 2, 1915. Age 44.

HENRY OSTHUES, Niagara University, afterward professor of anatomy in his Alma Mater; died in Brooklyn, N. Y., Apr. 4, 1915. Age 46.

1898 CHARLES B. BRAMAN, died in Clifton Springs, July 4, 1914. Age 53.

1903 FRANK JONES, died in Niagara Falls, N. Y., Aug. 4, 1914. Age 38.

1909 EDWARD D. CLARK, died in Syracuse, N. Y., Feb. 13, 1915. Age 27.

### CLASS REUNIONS.

The Class Reunions took place on Wednesday evening, June 2d. The Class of 1870, in charge of Dr. George E. Blackham of Dunkirk, N. Y., held its dinner at the Hotel Statler. Dr. Charles Cary arranged for the gathering of the class of 1875 at the Buffalo Club. The class of 1880 dined at the Hotel Statler, thirteen members of the twenty-five living attended and credit for the success of this gathering is due to Dr. Benjamin H. Grove. Dr. Edward Clark was chosen permanent secretary. Under the direction of Dr. Thomas F. Dwyer, the class of 1885 held its reunion at The Genesee and elected Dr. Jacob Goldberg permanent secretary. The class of 1890 held its 25th reunion at the Buffalo Club with Dr. J. F. Whitwell as master of ceremonies; Dr. Burt C. Johnson was elected permanent secretary. The banner dinner in point of attendance and enthusiasm was that of the boys of 1895,—Drs. Charles R. Borzilleri and John H. Daniels assembled 24 classmates at the Hotel Statler. Dr. Borzilleri was made permanent secretary. Drs. George W. Gorrill and James H. Carr managed the reunion of the class of 1900 which was held at the Hotel Lafayette, Dr. Gorrill being elected permanent secretary. The class of 1905 under the leadership of Drs. D. C. McKenney and Isaac Sernoffsky held its reunion at the Touraine Hotel. The members of the class residing in Buffalo entertained their out-of-town classmates with an attendance of 23. The idea of the in-town men entertaining their out-of-town classmates is a good suggestion for future class reunions. Dr. John

N. Flannery was elected permanent secretary. The baby reunion class (1910) had a theatre party for 14 at Shea's followed by a supper at the Hotel Statler. This class decided to offer a cup upon which is to be inscribed the name of the class having the largest attendance at the reunion each year. Dr. Clayton W. Greene was elected permanent secretary. The classes of 1894 and 1904 held their annual reunion as agreed last year.

### THE ANNUAL DINNER.

The annual dinner of our association which has become the social event of our meeting took place on Thursday evening, June 3rd, at the Hotel Statler. The attendance was over 200, the largest in our history. Our guests of honor were Drs. Joseph C. Beck of Chicago and Samuel J. Kopetzky of New York City. After a most excellent repast which was thoroughly enjoyed by all, the retiring president, Dr. George F. Cott, '84, introduced as toastmaster of the evening Dr. Thomas H. McKee, '98, and known to us all as "Tom McKee." Dr. Cott in the course of his remarks called to our attention the value and importance of alumni loyalty and stated what the graduates of other institutions had done for their Alma Mater. With the changing times he thought our alumni were showing greater interest and enthusiasm in Alma Mater from year to year. To dilate on Tom McKee's qualifications as toastmaster would require a volume. Suffice it to say that he acquitted himself in his usual graceful manner introducing each speaker fittingly. The toasts of the evening were directed largely to two purposes, viz., the retirement of Dr. Peter W. Van Peyma, '72, from the teaching of obstetrics and the consideration of the prospective Alumni Fund. Dr. James E. King, '95, under the caption "Our Nestor in Obstetrics" spoke very feelingly and eloquently on Dr. Van Peyma's ability as a teacher and the services rendered to the students of the past thirty years or more of his teaching as well as his splendid attainments in practical obstetrics. Special emphasis was put on the Obstetric Conference at which Dr. Van delighted all who were privileged to attend same for who of us can forget the noble naturalness as well as the simple directness used by our retiring master teacher in conducting this course? In responding, Dr. Van Peyma gave some of his "Observations and Recollections" whilst a student in his Alma Mater as well as in the clinics of Vienna, Dresden, Berlin and Leipsic, how the teaching of obstetrics had changed from didactic to clinical and the great pleasure it afforded him to impart knowledge to us. The class of 1915 through its President Dr. Dan S. Bellinger, presented a picture of Dr. Van Peyma, to the University bearing the following inscription:

**Peter W. Van Peyma, M. D., 1872.**
**Teacher and Clinical Professor of Obstetrics**
**1890-1915, when he retired.**
**Presented by the Class of 1915**
**In appreciation of his attainments**
**In the Practice and Teaching of Obstetrics.**

Dean Herbert U. Williams accepted this picture on behalf of the faculty, in an original poem.

The prospective **Alumni Fund** was discussed by Drs. Daniel P. Murphy, '02, of Elmira, N. Y., and Lee A. Whitney, '01, of Rochester, N. Y. "Dan" Murphy very eloquently directed our attention to the present needs of our Alma Mater and claimed that it was the duty of the Alumni to assume responsibility for the institution from which they graduated. He urged the raising of the Alumni Fund to which every alumnus should feel duty bound to contribute, for surely all owe more to the University than was paid to it in tuition. Dr. Whitney gave some very practical suggestions on the establishment of the fund and advised the following methods:

1. The payment of the contribution at one time; 2. The payment of one's contribution in annual installments, or, 3. An annual contribution by each alumnus of such amount as can be conveniently given. The latter will appeal to the younger men as it will permit them to increase their contribution if so desired with their increasing incomes.

The humorous address of the evening and yet one containing much homely philosophy was given by Dr. William House, '95, of Portland, Ore., who participated so largely in the general success of our meeting. The subject of his toast was "Medicine in the Wild and Woolly West." The Doctor cited many humorous experiences in his practice and teaching of medicine and showed that the status of our confreres on the Pacific coast is equal to that in the East. Singular as it may seem, the per capita proportion of physicians to the population is fully equal to that in the East. For the benefit of those who contemplated attending the Panama-Pacific Expositions at San Francisco and San Diego, Dr. House discussed the several routes of travel giving a very succinct and practical description of each.

Our Alumnae to the number of thirty or more graced our festive board and Dr. Maud J. Frye, '92, responded on their behalf.

The graduating class (1915) were installed to membership in this association by toastmaster McKee, each one assuming the obligation of loyalty to Alma Mater and promising to live in accord with the high ideals of our noble profession.

Dr. Clayton W. Greene, permanent secretary of the class of

1910, reported the action taken at their reunion in contributing a loving cup.

Dr. Julius Richter, '04, our efficient secretary, prepared a series of cartoons featuring some special fact relative to a number of our more active and energetic alumni. These were projected on a screen with a stereopticon and added largely to the levity of the evening. The decorations of the hall were also in charge of Dr. Richter.

The singing of college songs was rendered with even more spirit and vim than in years past. Special credit should be given to Dr. Norman L. Burnham, '96, for the success of this feature. As a chorister and song writer Norman certainly has no equal. The alumni prize song "The Bison is King of Them All," words and music by Dr. Herbert U. Williams, '89, was the hit of the evening.

Dr. Kauffman, retiring chairman of the executive committee announced the offering of two prizes for the best songs to be submitted by the alumni of any department of the U. of B. prior to January first, 1916,—the selection to be made by a committee of impartial judges and the songs to be rendered at the second annual dinner of the Associated Alumni to be held in Buffalo on the evening of University Day (February 22, 1916). Lest Dr. Williams again become the prize winner, our members are requested to begin at once so as to send in their contributions in due season.

### FRATERNITY REUNIONS.

These were held on Tuesday night, June 1st. The Nu Sigma Nu (I. C. I. chapter), Phi Rho Sigma (Alpha Omega Delta chapter) and the Omega Upsilon Phi fraternities entertained their members at their respective chapter houses.

### THE UNIVERSITY BISON.

The attention of the alumni is again directed to the U. of B. paper, **The University Bison,** which is published monthly and is edited and controlled by the student body. This paper contains a report of all University activities of both the graduate and undergraduate body and therefore deserves your support. Subscriptions can be sent direct to the business manager.

### COMPARATIVE STATEMENT OF ACTIVITIES FOR THE PAST FOUR YEARS.

| | 1912 | 1913 | 1914 | 1915 |
|---|---|---|---|---|
| Registration | 263 | 324 | 410 | 385 |
| Attendance at Annual Dinner | 130 | 175 | 200 | 225 |
| Balance in Treasury | $132 | $260 | $250 | $200 |

This statement shows a steady and healthy increase in the activities of our association from year to year and were it not for the meeting of the State Society coming six weeks prior to our own gathering, our attendance would have exceeded last year. The increased expenses incident on the organization of the District Alumni Associations account for the reduction in the balance in our treasury, we simply must have more dues paid in each year.

PLANS FOR THE COMING YEAR.

The executive committee in planning its arrangements for the ensuing year asks your favorable consideration of and hearty support in all of its efforts. There are many ways in which all can co-operate towards the increasing alumni activities.

1. ALUMNI CATALOGUE. This much promised and long expected book is now in the hands of the printer and will be mailed in **October.** It contains the new constitution and by-laws, a list of members by classes, as graduated from the University, a geographical and alphabetical index, and other data relative to the University and our Association.

2. LOCAL ALUMNI MEETINGS. (a) Chautauqua District Association, meeting at Olean, N. Y., October 21, 1915. (b) Central and Northern New York Alumni Association, meeting at Syracuse, N. Y., November 18, 1915. (c) Interstate Alumni Association, meeting at Elmira, N. Y., March 27, 1916. (d) Rochester District Association, meeting at Rochester, N. Y., April 13, 1916. (e) Metropolitan Alumni Association, consisting of the alumni residing in New York City and vicinity, to be organized in New York City on March 28, 1916.

3. ANNUAL DINNER OF THE ASSOCIATED ALUMNI (all departments) at Buffalo on University Day, (February 22, 1916).

4. FORTY-FIRST ANNUAL MEETING OF THE ASSOCIATION—Tuesday to Friday, May 30 to June 2, 1916. Arrangements are already under way to hold clinics on three day of the meeting, same to be given by the masters of medicine and surgery from the large medical centers. The program will excel all previous ones. The Harrington Lectures will be a special feature, the speaker in contemplation is one of the great authorities in his field of study and his subject will be of interest to all. At this meeting we celebrate the seventieth year of the foundation of our University which in itself is an important event. Whilst the officers are at all times ready and willing to do their utmost in planning the arrangements, it is absolutely essential to have the united co-operation of the entire alumni body in order that the meeting be a success. **Reunions** will be held by classes of '61, '66, '71, '76, '81, '86, '91, '96, '01, '06, and '11, the same to be in charge of respective class committees. Therefore, it behooves the members of these classes as well as the other alumni to plan their arrangements so as to be in Buffalo at the meeting, thereby showing their ever-increasing enthusiasm for and loyalty to Alma Mater.

Arrangements have been made with Dr. A. L. Benedict, '88, editor of THE BUFFALO MEDICAL JOURNAL in which this report is published to have an Alumni page wherein all announcements of this association will appear and any news items sent to the editor will be published therein.

